# Study protocol for ADHERE (Applying Directly observed therapy to HydroxyurEa to Realize Effectiveness): Using small business partnerships to deliver a scalable and novel hydroxyurea adherence solution to youth with sickle cell disease

**DOI:** 10.1371/journal.pone.0304644

**Published:** 2024-06-25

**Authors:** Joseph Walden, Lauren Brown, Sebastian Seiguer, Katie Munshaw, Joseph Rausch, Sherif Badawy, Patrick McGann, Savannah Winkler, Lisbel Gonzalez, Susan Creary

**Affiliations:** 1 Center for Child Health Equity and Outcomes Research, The Abigail Wexner Research Institute at Nationwide Children’s Hospital, Columbus, OH, United States of America; 2 emocha Mobile Health Inc. Doing Business as Scene and Scene Health, Baltimore, MD, United States of America; 3 Center for Biobehavioral Health, The Research Institute at Nationwide Children’s Hospital, Columbus, OH, United States of America; 4 Department of Pediatrics, College of Medicine, The Ohio State University, Columbus, OH, United States of America; 5 Division of Hematology, Oncology, and Stem Cell Transplant, Lurie Children’s Hospital of Chicago, Chicago, IL, United States of America; 6 Department of Pediatrics, Northwestern University Feinberg School of Medicine, Chicago, IL, United States of America; 7 Lifespan Comprehensive Sickle Cell Center, Providence, RI, United States of America; 8 The Warren Alpert Medical School of Brown University, Providence, RI, United States of America; PLOS: Public Library of Science, UNITED KINGDOM

## Abstract

Sickle cell disease (SCD) is an inherited blood disorder that affects approximately 100,000 Americans, primarily from underrepresented racial minority populations, and results in costly, multi-organ complications. Hydroxyurea, the primary disease-modifying therapy for SCD, is effective at reducing most complications; however, adherence to hydroxyurea remains suboptimal and is the primary barrier to clinical effectiveness. Video directly observed therapy (VDOT) has shown promise as an adherence-promoting intervention for hydroxyurea, yet previous VDOT trials were limited by high attrition from gaps in technology access, use of unvalidated adherence measures, and healthcare system limitations of delivering VDOT to patients. As such, we fostered a small business partnership to compare VDOT for hydroxyurea to attention control to address previous shortcomings, promote equitable trial participation, and maximize scalability. VDOT will be administered by Scene Health (formerly emocha Health) and adherence monitoring will be performed using a novel electronic adherence monitor developed to meet the unique needs of the target population. Adolescent and young adult patients as well as caregivers of younger patients (<11 years of age) will be recruited. In addition to visit incentives, all participants will be offered a smartphone with a data plan to ensure all participants have equal opportunity to complete study activities. The primary objectives of this pilot, multi-center, randomized controlled trial (RCT) are to assess retention and sustained engagement and to explore needs and preferences for longer-term adherence monitoring and interventions. This RCT is registered with the National Institutes of Health (NCT06264700). Findings will inform a future efficacy RCT applying VDOT to hydroxyurea to address adherence gaps and improve outcomes within this vulnerable population.

## Introduction

Sickle cell disease (SCD) is an inherited blood disorder that affects approximately 100,000 Americans, most of whom are from underrepresented racial minority populations [1]. Individuals with SCD are at risk for many acute and chronic complications, including vaso-occlusive pain crises, acute chest syndrome episodes, chronic kidney disease, pulmonary hypertension, and early mortality [2, 3]. The average cost of caring for a young child with SCD is >$14,000/year/child [4, 5] but this vastly underestimates SCD burden, as this does not include its impact on quality of life and because healthcare costs and utilization increase drastically in adolescence and young adulthood [4, 6–8]. Fortunately, effective disease modification early in the life-course has potential to attenuate this trajectory [9–11].

Hydroxyurea is the primary disease modifying therapy for SCD, with more than 40 years of evidence demonstrating remarkable effectiveness to reduce acute complications, mitigate many long-term costly SCD comorbidities, and improve survival [9–14]. Despite calls to increase hydroxyurea use among youth with SCD in the last decade, [15] their high rate of emergency department visits and hospitalizations have not significantly changed, [16] Non-adherence is a key reason for hydroxyurea’s limited impact, [17–22] as only 18–66% of youth achieve the level of hydroxyurea adherence seen in the pediatric trial that established its efficacy [18–20, 23]. Studies suggest that patient-level hydroxyurea adherence barriers include forgetting, competing priorities, and having to deal with SCD daily, while understanding hydroxyurea’s benefits, feeling better, and receiving support from others facilitate adherence [21, 24, 25].

To address the adherence gap, previous studies have explored various patient-centered interventions, including those involving community health workers, [26] text message reminders, [27] and other electronic health programs, such as video directly observed therapy (VDOT) [28–30]. VDOT is a technology-based version of directly observed therapy (DOT) which uses smartphones with cameras to make direct observation of medication administration as an adherence-promoting strategy more accessible and feasible. While results from early hydroxyurea VDOT studies were promising (e.g., mean hydroxyurea adherence by refill records increased among retained subjects) these studies were single-center and not randomized. Attrition was also high, primarily due to inconsistent personal access to smartphones and cellular data needed for VDOT. Although many adolescents with SCD own smartphones and are interested in app-based interventions to promote adherence, [31] reliable access to digital devices is especially relevant when considering VDOT as an adherence-promoting intervention for SCD, as underrepresented minorities are less likely to have skills to interface with mobile device technology and less likely to have access to high-speed internet than their White counterparts [32, 33]. Unfortunately, adherence appeared to decline after VDOT was discontinued, but the method used to measure adherence in these studies may not be valid [34]. Finally, clinical SCD teams’ ability to deliver VDOT at scale to the large population of youth taking hydroxyurea in the real-world setting was a potential challenge.

To address these challenges and promote equitable access and scalability of VDOT for youth with SCD in clinical practice, the study team partnered with Scene Health, Baltimore, MD (formerly emocha Mobile Health Inc.). Scene Health is a small business that offers a user-friendly mobile technology platform, a technology-enabled review service, extensive experience successfully engaging populations affected by both infectious and chronic diseases to report medication adherence with VDOT, [35–39] and commercial partnerships with health insurers. This partnership provides an opportunity to assess the feasibility and scalability of VDOT and electronic adherence monitoring in a pilot RCT.

### Objectives

Given prior challenges and the need for a successful and scalable hydroxyurea adherence solution to improve outcomes, the primary aim of this study is to assess retention and sustained engagement during a pilot randomized controlled trial (RCT) comparing VDOT for hydroxyurea delivered by Scene Health to attention control among youth with SCD. The secondary aim of this study is to use a novel adherence monitoring device to measure hydroxyurea adherence and explore needs and preferences for longer-term adherence monitoring and intervention. These findings will be used to inform a larger RCT to definitively determine if VDOT improves hydroxyurea adherence and outcomes.

## Methods

### Ethics

This study was initially approved by a single Institutional Review Board on 08/15/2023 (STUDY00003303) (S1 File) and is registered with the National Institutes of Health (NCT06264700). All participants will provide consent/assent. Relevant data will be made available at the conclusion of this study upon request. This trial includes all items on the Standard Protocol Items: Recommendations for Interventional Trials Checklist (S2 File).

### Design

**Fig 1:**
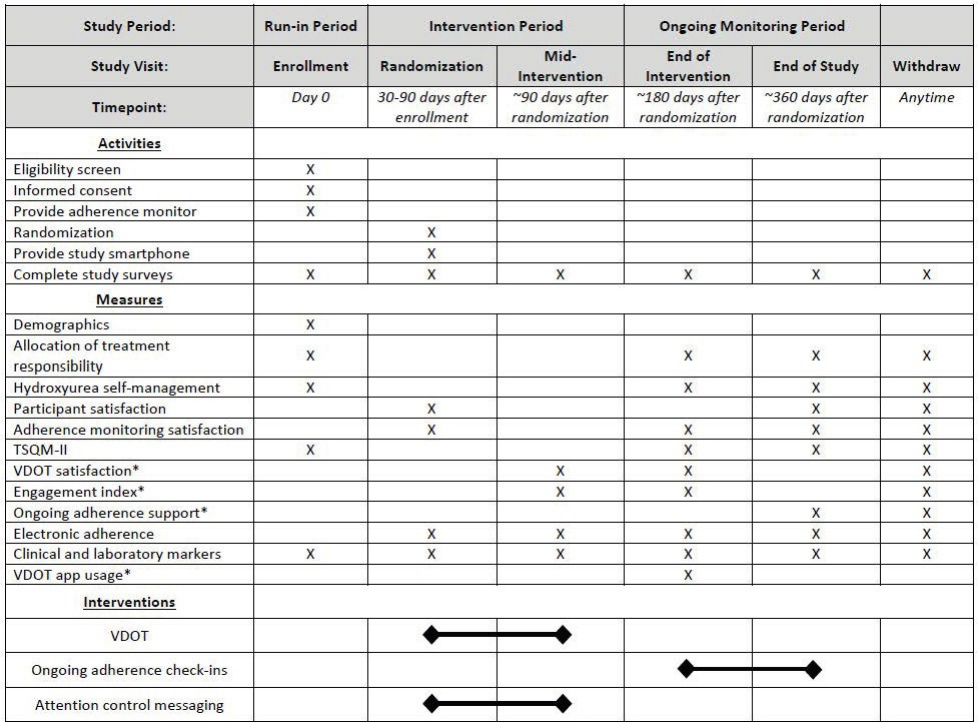
ADHERE SPIRIT schedule. *Only to completed by participants randomized to receive VDOT.

ADHERE (Applying Directly observed therapy to HydroxyurEa to Realize Effectiveness) is a pilot, multi-center, investigator blinded, RCT of caregivers of children with SCD and adolescents and young adults with SCD. After completion of at least 30-days of a run-in period, participants will be randomized 1:1 to the VDOT or attention control group. A 180-day intervention period will be followed by 180 days of ongoing adherence monitoring in both groups, during which time the VDOT group will receive monthly adherence updates from Scene Health (Fig 1).

### VDOT intervention

**Fig 2:**
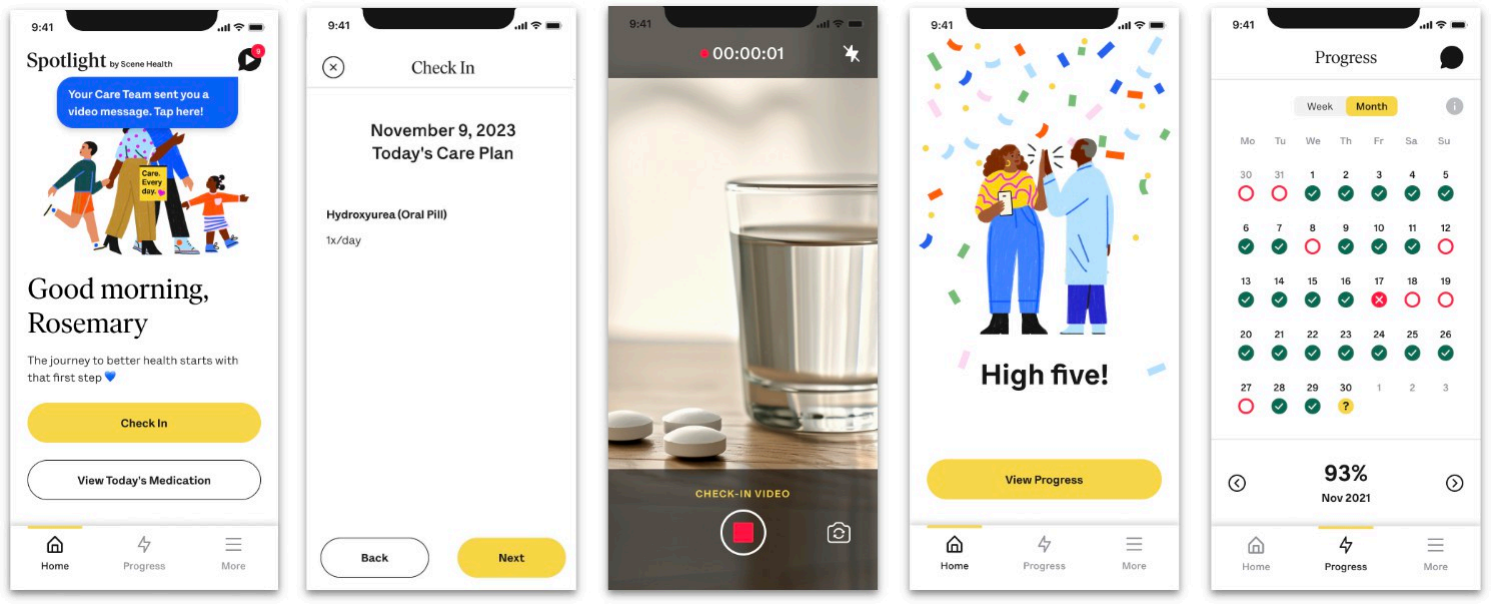
VDOT for hydroxyurea delivered via the “Spotlight by Scene Health” app. Reprinted from materials provided by emocha Mobile Health Inc., dba Scene Health, under a CC BY license, with permission from emocha Mobile Health Inc., original copyright 2024.

The VDOT intervention will be provided to all participants randomized to the VDOT group by Scene Health via a smartphone app (“Spotlight by Scene Health”) (Fig 2). The VDOT intervention consists of four components: scheduled medication reminders, participant-submitted hydroxyurea administration videos, personalized feedback from Scene Health’s trained staff, and monetary incentives to encourage medication adherence.

Scheduled reminders (“Hydroxyurea time!”) are sent via push notifications and/or text message to prompt hydroxyurea administration. Reminders are sent once per day in the evening, but participants can customize the timing, number, and type (i.e., push notification vs. text) of reminder messages based on their preferences. Participants record and submit daily videos of their/their child’s hydroxyurea administration. A daily video is required to be considered adherent to VDOT, even if hydroxyurea is suspended or prescribed on an atypical schedule (e.g., five times per week), except during hospitalizations. Hospitalized participants will be considered “adherent” on each hospital day. Personalized feedback is provided in real time to participants by trained Health Coaches at Scene Health to encourage or reinforce adherence, depending on their specific needs. This feedback is based on each participant’s use of the VDOT app and VDOT adherence rate, is unique to participants (e.g., including references to previous video submissions), and is primarily provided through a secure in-app chat feature. All default message content was created with input from patients who are prescribed hydroxyurea to ensure messages are applicable to the patient population. Lastly, participants are incentivized to achieve high adherence. They receive $15 for each month where they increase their adherence by ≥10% compared to the previous month or $30 for each month where they achieve ≥90% VDOT adherence.

The VDOT intervention for hydroxyurea was built using Scene Health’s existing platform and was customized to meet the unique needs of this study and patient population. The development process was iterative, taking place over the course of six months. It involved weekly meetings and collaboration between the study team and Scene Health researchers, product managers, and designers in addition to intermittent engagement with community partners, including patients with SCD. These meetings resulted in a detailed and comprehensive escalation protocol for common scenarios (e.g., medication refill requests) developed in collaboration with SCD clinicians, research team members, and the Scene Health program management and operations team to ensure safe, appropriate, and efficient communication between Scene Health, the research team, and the SCD clinical team will occur throughout the trial.

### Attention control

An attention control group will be used as the comparison group in this study to ensure that the effect of the VDOT intervention, and not the attention participants receive as a part of VDOT, can be evaluated. The attention control group receives an alternating daily health tip via text message at a time that is not common to administer hydroxyurea (e.g., the middle of the day) so that this attention is comparable to the level of attention that is uniformly delivered to all VDOT participants. These messages alternate from a bank of 24 messages that were adapted from the World Health Organization and Centers for Disease Control & Prevention tips for healthy living [40–45] that are appropriate for those with SCD (e.g., “Time to get moving! You should be active for at least 30 minutes each day.”).

### Passive electronic adherence monitoring device

The study team decided to use a novel passive electronic adherence monitoring device that is compatible with liquid medications formulations, including hydroxyurea, because this formulation is commonly prescribed to children. The device is designed to be user-friendly and self-explanatory, as it is a sleeve with a button that wraps around a variety of medication bottle sizes and formulations (e.g., capsule, liquid), with a battery that will last for duration of the study. Participants will be instructed to “click” the button each time they administer hydroxyurea throughout the study and to transfer the sleeve to the current medication bottle that they are using to administer hydroxyurea. Each button click will be securely transmitted to an online dashboard to allow for remote, long-term passive adherence monitoring. Digital adherence data collected by these devices will be used to track adherence throughout the study for participants in both groups and by Scene Health staff to inform their monthly coaching messages to VDOT participants during the ongoing monitoring period. The study team will also compare adherence data reported by the electronic device to video-observed adherence data collected from VDOT participants to determine if this device is a valid adherence monitor to be used in future studies.

### Setting, recruitment, participants

This study will recruit from three pediatric sickle cell centers located within academic medical centers, two in the Midwest and one in the Northeast. Recruitment will begin on approximately June 1, 2024. Eligible participants will be identified through queries of each institution’s clinical SCD database and will be approached during routine appointments, during hospitalization, or by telephone to describe the study assess interest in participating.

Youth (or caregivers of youth) with SCD (any genotype) aged 1–25 years who are receiving care at any of the study sites and who have been prescribed hydroxyurea for at least 180 days prior to enrollment will be eligible for this study. Those who are prescribed another disease-modifying medication (e.g., L-glutamine, voxelotor, or crizanlizumab) or who participated in a previous VDOT study will be excluded. Caregivers of youth aged 1–10 years with SCD will be enrolled as the participants for the entire study period, while adolescents and young adults aged 11–25 years with SCD will be enrolled themselves. All participants must speak English. Prior to any study procedures, informed consent will be obtained from adult study participants ≥18 years of age and from caregivers of participants <18 years old. Assent will be obtained for all participants 9–17 years of age.

We expect to approach ≤90 prospective participants (∼30 per site) to recruit a planned total sample of 60 participants. We aim to recruit a relatively even distribution of participants across sites and between caregiver participants and adolescent and young adult participants.

### Procedures

**Fig 3:**
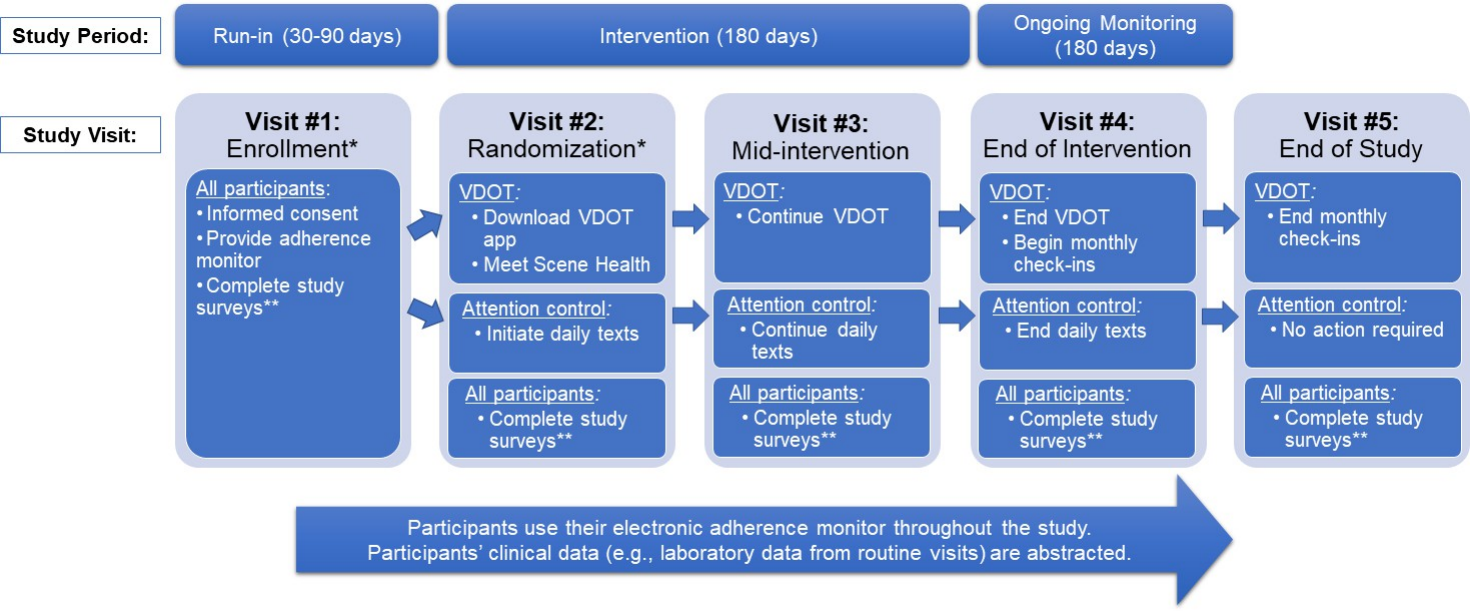
Overview of ADHERE study visits. *Visits #1 and #2 will occur in-person. Visits #3-#5 will be in-person or virtual, depending on participants’ clinical hematology visits. **Study surveys will measure key outcomes at various time points such as participant satisfaction with hydroxyurea and their trial experience.

The ADHERE trial will include a total of five study visits (Fig 3). Study visits will align with regularly scheduled hematology clinic visits whenever possible, with various study activities and surveys being completed at each visit. Study staff will be responsible for facilitating study visits, administering surveys, distributing study-related devices/technology, and abstracting clinical data. Scene Health will deliver VDOT during the intervention period and administer monthly check-ins to the VDOT group during the ongoing monitoring period. All participants will receive $20 for completing each study visit. If surveys cannot be completed in-person, participants will be able to complete them electronically.

#### Study visit #1: Enrollment

After informed consent, participants will receive their electronic adherence monitoring device. Study staff will administer scheduled surveys and instruct participants to use their electronic monitoring device to track each time they take/administer hydroxyurea at home.

#### Run-in period

The run-in period will begin after enrollment and last for at least 30 days for all participants. This period may last up to 90 days to allow for the coordination of study visits with regularly scheduled hematology visits when possible. Participants will be eligible for randomization if they use their electronic adherence monitoring device at least once during the run-in period.

#### Study visit #2: Randomization

At the second study visit, eligible participants will be randomized by study staff. Randomization is 1:1 by study site to either receive VDOT or attention control in randomly varying blocks of two and four. Study staff will administer scheduled surveys and screen participants for study-related and device-related issues. All participants, regardless of study arm assignment, will also be offered a smartphone with an unlimited data plan for use throughout the study to address the attrition seen in previous VDOT trials and technology access gaps in the United States [28, 29, 32, 33].

For participants randomized to VDOT, study staff will assist with VDOT initiation, including app download, account set-up, test video submission, and an introduction to the Scene Health team. For participants randomized to attention control, study staff will initiate the daily health text messaging software.

#### Intervention period

VDOT and daily health tips will begin the day after randomization and continue for 180 days. All participants will use their electronic adherence monitor throughout the intervention period.

#### Study visit #3: Mid-intervention

A study visit will occur approximately mid-way through the intervention period. Study staff will administer scheduled surveys and screen participants for study-related and technology-related issues at an in-person encounter or electronically if this is not possible.

#### Study visit #4: End of intervention

A study visit will occur approximately 180 days after the intervention period started. Study staff will administer scheduled surveys and screen participants for study-related and technology-related issues at an in-person encounter or electronically if this is not possible.

#### Ongoing monitoring period

After 180 days from randomization, VDOT or attention control messaging will end. Both groups will continue using their electronic adherence monitors for 180 more days. In addition, Scene Health staff will continue interacting with the VDOT group via monthly telephone calls and intermittent text messages to offer ongoing, personalized adherence support.

#### End of study visit

A final study visit will occur approximately 180 days after the ongoing monitoring period began. Study staff will administer scheduled surveys and screen participants for study-related and technology-related issues at an in-person encounter or electronically if this is not possible.

### Measures

Multiple subjective and objective measures will be captured to assess retention, sustained engagement, adherence, and the needs and preferences for longer-term adherence monitoring and intervention (Table 1).

**Table 1:** Data to be collected.

Tool	Description	Participant Group	Study Visit	Purpose
**Demographic Survey**	Self-reported age, gender, race, ethnicity, education level, and employment status.	All participants	Study visit #1	To describe the study population
**VDOT Satisfaction Survey [28, 46]**	15-item, Likert survey	VDOT only	Study visits #3 and #4	To understand acceptability and feasibility of VDOT and how to optimize it
**Engagement Index Survey [47]**	10-item, Likert-style survey	VDOT only	Study visits #3 and #4	To evaluate engagement with VDOT
**Allocation of Treatment Responsibility (ATR) Questionnaire [48, 49]**	18-item, Likert survey	All participants	Study visits #1, #4, and #5	To assess self-reported responsibility for hydroxyurea adherence-related tasks change with VDOT
**Treatment Satisfaction Questionnaire, Version II (TSQM) [50]**	11-item, Likert survey	All participants	Study visits #1, #4, and #5	To assess medication satisfaction, a key component of adherence
**Participant Satisfaction Survey [51]**	22-item, Likert survey with open-ended items	All participants	Study visit #5	To optimize study procedures for the efficacy RCT
**Adherence Monitoring Satisfaction Survey**	10-item, Likert survey with open-ended items	All participants	Study visits #2, #4, and #5	To evaluate acceptability of adherence monitoring
**Ongoing Adherence Support Survey**	8-item, Likert-style survey	VDOT only	Study visit #5	To evaluate the acceptability of longer-term adherence monitoring
**Electronic Medical Record Review**	Laboratory data clinical information, and hydroxyurea dosing	All participants	Study visits #1 - #5	To optimize study procedures and inform the efficacy RCT
**VDOT App Usage**	Frequency and time spent using the VDOT app	VDOT only	Study visits #1 - #5	To objectively measure VDOT engagement
**Adherence Monitoring Device**	Electronic adherence rate from the adherence monitoring device.	All participants	Study visits #1 - #5	To objectively measure adherence

### Data management plan

Data will be entered and stored in REDCap, a secure, web-based application designed for data capture in research. All data will either be collected directly from participants via surveys or entered by authorized study staff under the supervision of the principal and co-investigators. Separate adherence reports will be generated for all participants based on their passive electronic adherence monitoring device data and by Scene Health for VDOT participants. These adherence data will be stored separately but coupled with each participant via a unique study ID.

### Safety considerations

A data safety monitoring board affiliated with the single IRB will provide monitoring and oversight of this study, including the potential study-related risk for loss of confidentiality and medication-related risk for severe myelosuppression from increased hydroxyurea adherence and exposure. Additional aspects of participant safety are considered with Scene Health’s escalation protocol. This escalation protocol includes notifying the clinical team if participants report side-effects from the medication, if they observe any urgent health and safety concerns (e.g., patient hospitalized, dangerous living conditions), or if they observe system-level adherence barriers (e.g., lost medication). This allows the clinical team to be updated on important clinical information that may be disclosed during observers’ interactions with participants.

The “Spotlight by Scene Health” mobile app is HIPAA-compliant and allows for secure, temporary storage of encrypted videos on smartphones until a network connection is made to a secure server. Access to the app via smartphone is protected by participant login credentials. Videos submitted for the intervention are not viewable in a phone’s general media galleries and participants’ video data are deleted from the device as soon as receipt is confirmed by the server. Only approved project staff from the study sites and Scene Health will have access to participant data via the web portal and mobile app.

### Statistical analyses

#### Prior to statistical analysis

Continuous data will be described using means, medians, ranges, and standard deviations, while categorical data will be reported using frequencies. Should participants withdraw, their reason for withdrawal will be documented and data will be collected nearest to the time of withdrawal for those providing consent.

#### Formal analyses

*Aim 1*. Retention rates will be calculated for each group during each study period. A multi-method analysis approach will be used to assess engagement. Means, medians, ranges, and standard deviations will be calculated for quantitative items.

*Aim 2*. Participants’ needs and preferences for longer-term adherence monitoring will be evaluated using a multi-methods analysis approach. Means, medians, ranges, and standard deviations will again be calculated for quantitative items.

*Exploratory aims*. We will also explore electronic hydroxyurea adherence, laboratory values (e.g., hemoglobin, fetal hemoglobin), and clinical outcomes (e.g., acute care utilization) between groups.

#### Qualitative analysis

At least two trained research staff will perform inductive content analysis [52, 53] on open-ended survey items and report emergent themes by study group (VDOT vs. attention control) and participant type (adolescent/young adult vs. caregiver). Kappa scores will be calculated to report inter-rater reliability.

## Discussion

Despite hydroxyurea’s efficacy in clinical trials, [9–14] non-adherence remains a major barrier to improving outcomes in SCD [17–21]. VDOT is a promising intervention because it targets multiple patient-level adherence barriers (e.g., forgetting, competing priorities, dealing with SCD daily) and facilitators (e.g., understanding hydroxyurea’s benefits, feeling better, receiving support from others) that have been reported in people with SCD [21, 24, 25]. These study results will inform the design of our larger and more definitive future RCT. For instance, our recruitment rate will inform if our eligibility criteria need to be expanded to increase enrollment, while our adherence analyses could inform if our population may need to be modified or more selective to target the patients who are more likely to benefit from VDOT. Also, our participant feedback and survey data will build upon prior work outlining user preferences for technology-based interventions [31] and inform if this iteration of VDOT needs to be further modified to increase engagement and retention. Participant feedback will also inform how longer-term adherence monitoring and intervention will be integrated into the RCT.

### ADHERE promotes feasibility of and equitable access to VDOT

Previous trials utilizing VDOT were limited by high attrition rates due to lack of technology access [28, 29]. This multi-center pilot RCT addresses these limitations and challenges. The socioeconomic disparities and limited reliable access to technology [32, 33] that were previous barriers to study participation and retention are addressed by offering smartphone devices and cellular data. This emphasis on equity in clinical research will allow for a more sociodemographic heterogenous participant population and lead to more generalizable results. Although this approach includes additional costs to pay for these devices and data (<$1,000 per patient per year), the costs of providing trained Health Coaches are substantially less than the average cost of one uncomplicated admission for a young child with SCD [5]. Considering many children with SCD have more than one admission per year, [4, 54] but hydroxyurea, if consistently taken, reduces hospitalizations, [11] finding effective means to improve hydroxyurea adherence could be cost-saving. Furthermore, integrating the costs of VDOT and smartphone access into routine coverage for SCD could help prevent costly downstream disease-related complications may be sustainable if it proves acceptable and impactful.

### ADHERE emphasizes collaboration to maximize safety while promoting scalability and adherence

This approach also outlines a collaborative solution to safely offer an adherence-promoting intervention to a diverse population without increasing the burden on clinical care teams with limited resources [55]. For example, the escalation protocol was designed to identify common issues and scenarios that could arise during this patient-engaging intervention and outlines a pathway for efficient communication between all parties to maximize patient safety and address barriers (e.g., lost medication or incorrect medication administration) effectively in real time.

This protocol may also be informative for the scalability of other technology-based interventions involving large amounts of data. For example, recent studies exploring the impact of wearable technology on the identification and treatment of atrial fibrillation have identified the importance of filtering out excess data to maximize the proportion of cases requiring intervention that are escalated to the clinical team [56–62]. We have considered these factors here to ensure that the Scene Health team is empowered to engage with participants and assess their adherence patterns while involving the clinical team, when necessary, without overburdening them. This partnership may eventually result in Scene Health delivering real-time adherence data to SCD clinicians so that they can use it to inform their clinical management, as recent adherence work in sleep apnea suggests that data delivered from wearable technology to clinicians offers more granular data that they can use to inform clinical management [63]. Clearly, there are several ongoing efforts across various medical fields to leverage real-time data from technology to report adherence and drive interventions [61–67]. We have designed this protocol to efficiently and safely integrate such data to promote scalability of a multi-faceted, technology-driven intervention.

### Anticipated outcomes and future directions

Anticipated outcomes from this study are that VDOT will be acceptable and result in sustained retention and engagement with a centrally-delivered adherence intervention. These results will be used to inform the definitive efficacy RCT of VDOT for hydroxyurea. As disease-modifying medications for SCD continue to emerge, [68–71] establishing an effective intervention to maximize adherence is especially relevant as it could inform strategies to expand such interventions to these newer medications as well.

## Supporting information

S1 File Institutional review board approved protocol. (DOCX)

S2 File SPIRIT checklist. (DOC)
