# Supplementary material for: Study protocol for ADHERE (Applying Directly observed therapy to HydroxyurEa to Realize Effectiveness): Using small business partnerships to deliver a scalable and novel hydroxyurea adherence solution to youth with sickle cell disease
Source: PLoS One. 2024 Jun 25;19(6):e0304644. doi: 10.1371/journal.pone.0304644 (PMC11198815; doi:10.1371/journal.pone.0304644)
Supplement: S1 File — (DOCX) [file pone.0304644.s001.docx]

**PROTOCOL TITLE:**

ADHERE (Applying Directly observed therapy to HydroxyurEa to Realize Effectiveness)

**PRINCIPAL INVESTIGATOR:**

Name: Susan Creary, MD, MSc

Department: Center for Child Health Equity and Outcomes

Telephone Number: 614-722-3563

Email Address: susan.creary@nationwidechildrens.org

**VERSION NUMBER/DATE:**

Version: 4

Date: 02/16/2024

**Revision History**

| **Version Date** | **Summary of Changes** | **Consent Change?** |
| --- | --- | --- |
| 07/21/2023 | Updated enrollment numbers, study timelines, and updated items in sections 27 and 30, per IRB recommendations. | Yes |
| 08/14/2023 | Updated section 28 per recommendations | No |
| 02/16/2024 | 1. Updated name and information about the electronic monitoring device  2. Updated eligibility criteria to allow for enrollment of young adults up to 25-years-old  3. Included reference to identifiable tracking sheet (section 6.0 and section 27.0)  4. Included references to face ID protection for scene health app (spotlight)  5. clarified participant information that will be needed for scene health to carry out the intervention | Yes |
|  |  |  |
|  |  |  |

# Study Summary

| **Study Title** | ADHERE (Applying Directly observed therapy to HydroxyurEa to Realize Effectiveness) |
| --- | --- |
| **Study Design** | A pilot, multi-center, investigator-blinded, randomized controlled trial |
| **Primary Objective** | To assess retention and sustained engagement during a pilot RCT comparing video directly observed therapy (VDOT) to attention control |
| **Secondary Objective(s)** | To explore needs and preferences for longer-term adherence monitoring and intervention |
| **Research Intervention(s)/ Investigational Agent(s)** | VDOT |
| **IND/IDE #** | Not applicable |
| **Study Population** | Adolescents with sickle cell disease (SCD) and caregivers of young children (<11 years) with SCD |
| **Sample Size (finite #, no estimates)**  **(Other sites + Local = TOTAL)** | 60 across all sites. We will enroll at least 20 adolescents across all sites and at least 20 caregivers across all sites. The remaining 20 participants enrolled across sites will be either adolescents or caregivers. |
| **Study End Date** | The study will close five years after the approval date of this IRB. |
| **Study Specific Abbreviations/ Definitions** | VDOT video directly observed therapy  SCD sickle cell disease  RCT randomized controlled trial  EM ( electronic adherence monitors) |

# Objectives

|  |
| --- |
| The *primary objective* of this study is to conduct the ADHERE (Applying Directly observed therapy to HydroxyurEa to Realize Effectiveness) trial, a pilot, multi-center, investigator-blinded, randomized controlled trial (RCT) to inform the definitive RCT.  Our primary aim is to assess retention and sustained engagement during a pilot RCT comparing VDOT, a promising hydroxyurea adherence-promoting intervention, to attention control. We hypothesize that ≥80% of participants on each arm of the pilot RCT will be retained and VDOT randomized participants will score highly on engagement measures.  Our secondary aim is to explore needs and preferences for longer-term adherence monitoring and intervention. We hypothesize that ongoing electronic adherence monitoring and intermittent encouragement from the VDOT team will be acceptable and promote sustained adherence behavior change.  We will also explore the impact of VDOT on the adherence, clinical, and behavioral outcomes. |

# Background

|  |
| --- |
| Sickle cell disease (SCD) is an inherited blood disorder that affects approximately 100,000 Americans. The average cost of caring for a young child (<11 years) with SCD is >$14,000/year and half of these children have ≥1 hospitalization/year, costing an average of >$17,000/admission. These numbers, however, vastly underestimate SCD burden as healthcare costs and utilization increase drastically in adolescence and young adulthood. Fortunately, improved care early in the life-course has potential to attenuate or disrupt this trajectory.  Hydroxyurea is the primary disease modifying therapy for SCD. Clinical trials show that it can reduce acute complications and mitigate many long-term costly SCD comorbidities. Despite increasing hydroxyurea use among youth with SCD in the last decade, their high rate of emergency department visits and hospitalizations remain unchanged. Non-adherence is a key reason for hydroxyurea’s limited impact. Only 18-66% of youth achieve the level of hydroxyurea adherence seen in the pediatric trial that established its efficacy.  We previously conduced a single-center, single-arm, six-month study of a hydroxyurea adherence intervention, called video directly observed therapy (VDOT), in patients aged 1-19 years. VDOT was delivered by smartphones and an email platform. VDOT included reminder alerts, feedback from study staff to encourage adherence, and monthly monetary incentives for achieving adherence goals. Results were promising; Mean hydroxyurea adherence (by refill records) increased from 59% at baseline to 82% among retained subjects. Yet, attrition was higher than anticipated and engagement with the intervention was not thoroughly assessed. Aspects of the project, including the method used to measure adherence and the non-randomized design limited the ability to definitively conclude that VDOT improves adherence. Finally, adherence declined after VDOT was abruptly discontinued and suggested that additional monitoring and encouragement after VDOT may be needed to promote lasting adherence behavior change. Participant feedback and experience from that study prompted us to partner with Scene Health, a small business that has a user-friendly VDOT platform and app and experience in successfully engaging and providing VDOT to other chronically ill populations. This partnership has potential to overcome the challenges that led to high attrition and to optimize VDOT’s impact. We were also able to partner with a company to develop a device unique to this trial’s needs that we will use to measure hydroxyurea adherence.  This project will provide the data needed to successfully complete the efficacy RCT that will aim to show that VDOT is effective and can translate hydroxyurea from a therapy that is effective in clinical trials to an effective therapy in clinical practice. This will enhance its attractiveness to payors to fund to reduce healthcare costs in this population. |

# Study Endpoints

|  |
| --- |
| To assess retention and sustained engagement during a pilot RCT comparing VDOT, we are using the following endpoints:  1. Assess recruitment (number of eligible and number enrolled) and retention to each phase of the study (e.g., run-in, intervention, and ongoing monitoring periods)  2. Use subjective surveys (e.g., VDOT satisfaction survey, the engagement index) and objective data from VDOT app to measure and assess intervention engagement and satisfaction  To explore needs and preferences for longer-term adherence monitoring and intervention, we use subjective surveys (e.g., adherence monitoring satisfaction survey, ongoing adherence support survey) and objective electronic adherence measures (electronic and VDOT measured adherence) to explore needs and preferences for prolonged monitoring and intervention  We will explore the adherence, clinical, and behavioral outcomes using the following outcomes:  1. Electronic and video observed hydroxyurea adherence  2. Laboratory markers  3. healthcare utilization  4. chronic end-organ damage  5. treatment satisfaction  6. adherence perception  7. satisfaction with the intervention  8. satisfaction with adherence monitoring  9. Satisfaction with study participation |

# Study Interventions/ Investigational Agent

|  |
| --- |
| VDOT is an adherence-promoting intervention that involves a healthcare worker partnering with an adolescent with SCD (or caregiver of a young patient with SCD) and observing the patient administer their hydroxyurea. VDOT includes:  1) automated short messages (e.g. “Hydroxyurea Time!”) up to four times daily to participants at their preferred time to remind patients/caregivers to administer their hydroxyurea,  2) video observed adherence of hydroxyurea administration via smartphone capture and upload,  3) personalized feedback messages via text message, email, and in-app chat messages to participants to encourage, motivate, and/or reinforce adherence from the Scene Health staff,  4) monetary incentives for each month that ≥90% hydroxyurea adherence or a ≥10% increase in adherence is achieved.  If hydroxyurea is suspended (i.e. for myelosuppression) or prescribed on an atypical schedule (i.e. five days/week instead of daily) participants are still instructed to submit a video daily, but videos are not required during hospitalizations to limit burden on ill patients.  VDOT is delivered by Scene Health and its staff. This is because Scene Health has:  1) a user-friendly VDOT platform and app that streamlines VDOT delivery and that have been used to improve adherence in other chronically ill populations  2) staff with experience building rapport, engaging patients, and encouraging adherence virtually and customize the frequency of their communication with each participant depending on their adherence and the platform allows staff to interact with patients through a variety of modes, including in-app messaging, recorded videos, and the “video-back” feature. While the messages can be customized, there is also a message bank covering common scenarios that can be sent to address common adherence barriers, and celebrate adherence streaks.  3) ability to interface with the clinical team if needed using an established escalation protocol but remains separate from the clinical team to prevent overburdening this team with adherence oversight. The escalation protocol includes notifying the clinical team if patients report side-effects from medication, if they observe an urgent health and safety concerns (e.g., patient hospitalized, dangerous living conditions), or if they observe system-level adherence barriers (e.g., lost medication). Per the escalation protocol, Scene Health can escalate to the clinical team depending on the severity and type of concern. This allows the clinical team to be updated on important information that may be disclosed during observers’ interactions with participants.  4) a web platform and mobile app that is HIPAA compliant. This system uses virtual communication (e.g., asynchronous video technology and secure 2-way in-app messaging) to support and encourage adherence through timely feedback and positive reinforcement. The system allows for secure, temporary storage of encrypted videos on smartphones until a network connection is made to a secure server. Access to the app is protected by a secure username/password, PIN, or face ID login. Videos are not viewable in a phone’s general media galleries. If a device with the app were lost or stolen, the app itself could not be accessed without a username and password. Data sent from mobile devices are encrypted on the device as soon as they are collected by the web portal. Patients’ video data are deleted from the device as soon as receipt is confirmed by the server. Only approved project staff from the study sites and Scene Health have access to patient data via the web portal and mobile app.  5) ability to provide a period of ongoing adherence support after intensive VDOT is provided. By combining review of the electronic adherence monitor (EM) and Scene Health platforms, Scene Health staff will make monthly telephone calls and provide text messaging during the ongoing phase of management. This integration will provide Scene Health staff with VDOT participants’ electronic adherence data from their EM devices and the ability use these data to inform the content of their telephone calls and messages.  Adherence monitoring will be completed using an electronic device because it will:   1. attach directly to the medication bottles that are dispensed from the pharmacy, including liquid medication bottles, and will be easily transferable to new medication bottles and transportable if/when patients travel. 2. Upload adherence data continuously via the cloud to a secure dashboard that is accessible to the Scene Health team. This will prevent participants from having to bring in their device to download the adherence data.   Participants will be notified prior to consent that the intervention and the electronic adherence monitoring will be delivered by outside companies and that the identifiable information provided to these companies will be limited to only that required to provide the intervention (see below). |
|  |
| - - - Drug Device Handling: |
| Participants will use their home-supply of hydroxyurea for this study. Hydroxyurea is FDA-approved for use in children and adults with SCD. |
|  |
| - - - If the drug is investigational (has an IND) or the device has an IDE or a claim of abbreviated IDE (non-significant risk device), include the following information: |
| Not applicable. Hydroxyurea is FDA-approved. |

# Procedures Involved

|  |
| --- |
| This is a multi-center, investigator-blinded, pilot RCT of caregivers of young children with SCD and adolescents with SCD. After a run-in period, subjects are randomized to either six-months of VDOT or attention control, and then will enter a six-month ongoing monitoring period. Outcomes are assessed during each study period. |
|  |
| **Recruitment**  The investigators will use their local clinical SCD databases to identify prospective participants and have an advertisement page available for clinic staff to pass out to patients and families with basic study information. Prospective participants will be approached during hospitalizations and during routine hematology and non-hematology appointments for participation. If a prospective participant has an upcoming scheduled appointment, we may attempt to call them ahead of this visit to inform them that they may be eligible and that they can come early to their clinical appointment to facilitate study enrollment.  **Run-In**  Following informed consent, enrolled subjects complete baseline assessments. If unable to complete all of these assessments at the enrollment visit, they will be able to complete them via electronic link.  Participants will receive their electronic adherence monitors (EM) and instructions for how to use these devices. Technical support for how to use these devices will be offered and provided by the research team if needed. Enrolled participants who complete at least 30 days of the run-in period and use their EM at home at least once in the run-in period will be eligible for randomization.  **Randomization**  Subjects meeting criteria will complete their randomization visit typically during routine hematology visits, but these visits can also be completed during a hospitalization, if criteria are met prior admission. Randomization is 1:1 to VDOT or attention control groups by study site, and randomly varying blocks of size 2 and 4 will be selected with equal probability within each site. The randomization scheme within each stratum will be generated using a pseudorandom number generator in the statistical software package R.  **Smartphones and Data**  After randomization, all participants will be offered a smartphone and unlimited data plan to use during the remainder of the study. They will be instructed to continue to use their EM throughout the entire study.  **VDOT Participants** A study research staff member (not the PI or Co-I’s) will introduce the participant to the Scene Health staff who will guide participants through VDOT. A test video will be created by the participant and submitted for approval before the adherence video monitoring begins. The participants’ hydroxyurea dosing schedule will be entered into the app by the research staff and will be updated by these staff after their routine hematology visits and/or hospitalizations. Participants will receive VDOT for 180 days, beginning the day after randomization. After that time, they will start a 180-day ongoing monitoring period. During the ongoing monitoring period, VDOT participants will receive monthly telephone calls and intermittent text messages from the Scene Health staff to encourage ongoing adherence. The Scene Health staff will access the electronic adherence platform and use the electronic adherence data on this platform to inform their communications. At the end of the ongoing monitoring period data plans will be discontinued but study devices (EM and smartphones) will not be collected. Broken, lost, or stolen EM and/or smartphones will not be replaced, but participants will be able to remain enrolled and to complete study visits.  **Attention Control** **Participants** will receive an automated, daily, short health or safety tip alert about their child (e.g. “An apple a day”) during the 180 day intervention period on their smartphone that is unrelated to hydroxyurea adherence. After the 180-day intervention period, participants complete a 180 day ongoing monitoring period where they will no longer receive these messages but they will be reminded to continue to use their EM. At the end of the ongoing monitoring period, data plans will be discontinued but study devices (EM and smartphones) will not be collected. Broken, lost, or stolen smartphones will not be replaced, but participants will be able to remain enrolled and to complete study visits.  **Survey Completion** VDOT and Attention Control Participants will complete surveys at multiple study visits, but if a participant is unable to complete all of these assessments at an in-person visit, they will be able to complete them via electronic link.  **Investigator Blinding** Investigators will be blinded to reduce the potential for differential treatment post-randomization that could limit the ability to determine if VDOT improves hydroxyurea adherence.  **Study Visits** Survey data will be collected will occur at enrollment (day 0), randomization (30-100 days after enrollment), mid-intervention (60-120 days post-randomization), end of the intervention period (120-210 days post-randomization), at the end of the observation period (210-390 days post-randomization and at least 90 days after intervention ends), or when a participant withdraws (within 30 days of withdraw). While the enrollment and randomization visits need to occur in-person so devices can be delivered to participants, subsequent visits will coincide with routine hematology visits when possible but also be able to be completed at home electronically. Participants will receive $20 for completing each study visit. |
|  |
|  |
| **Potential Risks**  The potential risks to participants include:  1) potential loss of confidentiality  2) myelosuppression from increased hydroxyurea exposure from increased adherence.  **Protection Against Risks**  *1.* Informed Consent and Assent: Prospective participants will be informed about the potential risks that are involved with participating in this study, including loss of confidentiality. Furthermore, participants will be informed that the intervention will be delivered by app by Scene Health and an electronic device will be used to collect the electronic adherence data. After confirming eligibility and reviewing study procedures, a trained research assistant will obtain informed consent and assent (when applicable).  *2.* Loss of confidentiality: All clinical, laboratory, and survey data will be kept secure and de-identified and coded by study identification number, rather than by personal identifiers to prevent confidentiality breaches. A tracking sheet will be the only link between identifiable and de-identified data and will be stored on the hospital’s secure research drive. They will be informed that adherence data will be stored by study ID number on the secure EM platform and for those randomized to VDOT, they will be informed that the Scene Health app has a data security system and is an established HIPAA compliant app. Also, for those randomized to VDOT, the Scene Health app allows for secure, temporary storage of encrypted videos on smartphones until a network connection is made to a secure server. Access to the Scene Health app is protected by a secure username/password,PIN, or face ID login. Videos are not viewable in a phone’s general media galleries. If a device with the Scene Health app were lost or stolen, the Scene Health app itself could not be accessed without a username and password. Data sent from mobile devices are encrypted on the device as soon as it is collected. Video data are deleted from the device as soon as receipt is confirmed by the server. Only approved Scene Health project staff and study staff will have access to patient data via the companion web portal.  3. Myelosuppression: Hydroxyurea will be prescribed as standard of care during the study. Mild myelosuppression is expected and intended with hydroxyurea treatment, and it is not unusual for patients to have to temporarily discontinue hydroxyurea or to have their prescribed dose of hydroxyurea adjusted. There is a potential risk that increased hydroxyurea exposure from increased hydroxyurea adherence could result in severe myelosuppression. To minimize this risk, children will be dosed and prescribed hydroxyurea per standard institutional hydroxyurea guidelines and their clinician’s discretion. These guidelines recommend assessing self-reported adherence to hydroxyurea and obtaining blood work at least every three months to monitor for myelosuppression so that the prescribed hydroxyurea dose can be adjusted accordingly. The PI will review all severe myelosuppression events during the study (defined as an absolute neutrophil count <500/µL^3^, thrombocytopenia <50,000 µL^3^, and/or hemoglobin <7 g/dL with an absolute reticulocyte count <50×10^3^/μL^3^) for relatedness and expectedness to the intervention and these events will also be independently reviewed by the DSMB.  4. *Vulnerable Subjects:* We will enroll 60 subjects (~30 adolescents and ~30 caregivers) on the pilot RCT. The rationale for including adolescents as that these individuals are gaining autonomy and disease self-management skills and this intervention could help to support them.  We will enroll up to 60 subjects across all sites. We will enroll at least 20 adolescents across all sites and at least 20 caregivers across all sites. The remaining 20 participants enrolled across all sites will be either adolescents or caregivers. |
|  |
| - What data will be collected during the study and how that data will be obtained. |
| a) Clinical and laboratory data on adolescent participants who enroll and on the children of the caregiver participants who enroll will be abstracted from the electronic medical records. This data will be collected as de-identified and stored within the secured REDCap database.  b) An identifiable subject list will be kept separate from the REDCap database to track enrollment and key study milestones. This list will be coded by study ID and will include the following identifiers: participant name, MRN, DOB, phone number, email address, mailing address, and study visit dates. This will be the only link to de-identified data and will be securely stored on the hospital’s protected research drive which is only accessible by approved study staff.  c) Self and proxy-reported survey data will be collected electronically and stored as de-identified data within the secured REDCap database.  d) EM adherence data will be collected electronically from participants’ devices and stored as de-identified data on a secure electronic adherence device dashboard that is password protected and will only be accessible to the research and Scene Health staff.  e) Subjects randomized to VDOT will submit daily videos of themselves (or of their children) administering hydroxyurea using the secure VDOT app. The Scene Health team will have access to the minimal identifiable subject information needed to administer the intervention (e.g., participant name, DOB, telephone number, and email). The Scene Health platform has an established HIPAA compliant mobile app.  f) Data on VDOT subjects use of the VDOT app may also be collected (e.g., number of times that they access the App, amount of time that they spend on the App daily, level of interaction with Scene Health staff through in-app chat). |
|  |
| - If there are plans for long-term follow-up (once all research related procedures are complete), what data will be collected during this period. |
| Not applicable. There are no plans for long-term follow-up after study procedures are completed. |
|  |
| - For Humanitarian Use Device (HUD) uses provide a description of the device, a summary of how you propose to use the device, including a description of any screening procedures, the HUD procedure, and any patient follow-up visits, tests or procedures. |
| Not applicable. The intervention is not a HUD. |

# Study Timelines

| - Describe: - The duration of an individual subject’s participation in the study. - The duration anticipated to enroll all study subjects. - The date for the investigators to complete this study (complete primary analyses, study closure in eIRB2). |
| --- |
| **Duration of an individual subject’s participation**  ~13-15 months (1-3 month run-in, 6 month intervention, 6 month observation)  **Anticipated duration to enroll study subjects**  6 months from study launch  **Anticipated date for study completion**  12/2028 |

# Inclusion and Exclusion Criteria

|  |
| --- |
| Adult caregivers will be eligible for the pilot RCT if they:  1) are English speaking  2) have a child who is 1-10 years of age with SCD (any genotype) at enrollment who has been prescribed hydroxyurea for ≥180 days prior to enrollment.  Adolescents will be eligible for the pilot RCT if they:  1) English speaking  2) are 11-25 years of age at enrollment and will be followed at the same clinical site for the duration of the study period  3) have SCD (any genotype) and have been prescribed hydroxyurea for at least the 180 days prior to enrollment.  Adolescents and caregivers who participated (or whose child participated) in any previous VDOT study will be excluded. Also, adolescents and caregivers of children receiving additional SCD modifying treatments (e.g., voxelotor or L-glutamine) will be excluded. Caregivers of multiple children meeting criteria will only be able to enroll on the study for one child.  Adolescents and caregivers of children who discontinue hydroxyurea or care at their site or inform the team they do not want to continue to participate will be withdrawn. |
|  |
| - Indicate specifically whether you will include or exclude each of the following special populations: (You may not include members of the above populations as subjects in your research unless you indicate this in your inclusion criteria.)   - Adults unable to consent   - Individuals who are not yet adults (infants, children, teenagers)   - Pregnant women   - Prisoners |
| Adults unable to consent will be excluded.  Caregivers will be required to consent for adolescents ages 11-17 years who enroll, and these adolescents will be required to provide assent.  Caregivers of children ages 1-10 years of age at enrollment will be the participants. Children whose caregivers enroll and are ≥9 years will be required to assent to their caregiver’s participation since they will be involved in the intervention.  Pregnant caregivers may enroll. Eligibility for pregnant caregivers will be the same as for all other caregivers.  Pregnant adolescents and young adults are instructed to discontinue hydroxyurea during pregnancy, therefore they will not be eligible.  Prisoners will be excluded. |

# Vulnerable Populations

| If the research involves individuals who are vulnerable to coercion or undue influence:   - Check the box for the vulnerable population(s) involved. - Review the appropriate checklist (do *not* include in submission). - Describe additional safeguards included to protect their rights and welfare. |
| --- |
| Research involving pregnant women, reference “CHECKLIST: Pregnant Women (HRP-412)” |
| Pregnant caregivers will be able to enroll as there is no direct risk to the fetus. In addition to receiving the intervention or attention control, the other study activities (completing surveys and receive smartphones and data plans) do not pose a risk to the fetus.  Pregnant adolescents will be excluded, as hydroxyurea is discontinued during pregnancy. |
|  |
| The research involves neonates of uncertain viability or non-viable neonates, review “CHECKLIST: Neonates (HRP-413)” or “HRP-414 – CHECKLIST: Neonates of Uncertain Viability (HRP-414)” to ensure that you have provided sufficient information. |
| Not applicable |
|  |
| The research involves prisoners, review “CHECKLIST: Prisoners (HRP-415)” to ensure that you have provided sufficient information. |
| Not applicable |
|  |
| The research involves persons who have not attained the legal age for consent to treatments or procedures involved in the research (“children”), review the “CHECKLIST: Children (HRP-416)” to ensure that you have provided sufficient information. |
| This study involves children under 18 years but poses no greater than minimal risk. This is because the only two risks include: 1) potential loss of confidentiality and 2) myelosuppression from increased hydroxyurea exposure from increased adherence. Potential loss of confidentiality is an inherent risk ordinarily encountered in daily life or during the performance of routine physical or psychological examinations or tests. Additional safeguards (see section 6.0 above) are in place to limit this risk.  Furthermore, the risk of myelosuppression from increased hydroxyurea exposure from increased adherence to the prescribe dose can also be ordinarily encountered in daily life as standard clinical practice includes assessing hydroxyurea adherence and providing counseling or possible interventions to optimize adherence. Also, this risk is minimized as there are standard safety guidelines for monitoring and adjust hydroxyurea doses (see section 6.0 above).  Consent will be obtained from participants (both caregivers and patient participants) ≥18 years or the. Assent for adolescent participants ages 11-17 years will be required. |
|  |
| The research involves cognitively impaired adults, review “CHECKLIST: Cognitively Impaired Adults (HRP-417)” to ensure that you have provided sufficient information. |
| Not applicable. |

# Local Number of Subjects

| - - Indicate the total number of subjects to be accrued locally.   - If applicable, distinguish between the number of subjects who are expected to be enrolled and screened, and the number of subjects needed to complete the research procedures (i.e., numbers of subjects excluding screen failures.) |
| --- |
| We anticipate approaching ≤30 subjects about the study locally and enrolling about 20 subjects locally. Of those 20 local participants, we anticipate ≥16 of them to be randomized to either VDOT or attention control. We also aim to have approximately half of subjects who are screened, enrolled, and randomized to be caregivers and half to be adolescent/young adult patients. |

# Withdraw of Subjects

|  |
| --- |
| Participants who (or whose children) discontinue hydroxyurea or care at the study site or inform the research team that they do not want to continue to participate will be withdrawn.  Participants will also be able to withdraw for any reason and will be informed that they must do so in writing. Whenever possible, the reason for withdrawal will be documented. If participants who drop-out provide consent, minimal data (i.e. laboratory studies) will be collected nearest the time of the research variables of interest. We will attempt to have participants complete withdrawal survey measures within 30 days of their withdrawal. |

# Risks to Subjects

|  |
| --- |
| **Potential Risks**  The potential risks to participants include:  1) potential loss of confidentiality  2) myelosuppression from increased hydroxyurea exposure from increased adherence. |
|  |
| - - Describe what actions will be taken to minimize the risks (listed above). |
| ***Protection Against Risks***  *1.* Informed Consent and Assent: Prospective participants will be informed about the potential risks that are involved with participating in this study, including loss of confidentiality. Furthermore, participants will be informed that the intervention will be delivered by app by Scene Health and an electronic device will be used to collect the electronic adherence data. After confirming eligibility and reviewing study procedures, a trained research assistant will obtain informed consent and assent (when applicable).  *2.* Loss of confidentiality: All clinical, laboratory, and survey data will be kept secure and de-identified and coded by study identification number, rather than by personal identifiers to prevent confidentiality breaches. They will be informed that adherence data will be stored by study ID number on the secure EM platform and for those randomized to VDOT, they will be informed that the Scene Health app has a data security system and is an established HIPAA compliant app. Also, for those randomized to VDOT, the Scene Health app allows for secure, temporary storage of encrypted videos on smartphones until a network connection is made to a secure server. Access to the Scene Health app is protected by a secure username/password, PIN, or face ID login. Videos are not viewable in a phone’s general media galleries. If a device with the Scene Health app were lost or stolen, the Scene Health app itself could not be accessed without a username and password. Data sent from mobile devices are encrypted on the device as soon as it is collected. Video data are deleted from the device as soon as receipt is confirmed by the server. Only approved Scene Health project staff and study staff will have access to patient data via the companion web portal.  3. Myelosuppression: Hydroxyurea will be prescribed as standard of care during the study. Mild myelosuppression is expected and intended with hydroxyurea treatment, and it is not unusual for patients to have to temporarily discontinue hydroxyurea or to have their prescribed dose of hydroxyurea adjusted. There is a potential risk that increased hydroxyurea exposure from increased hydroxyurea adherence could result in severe myelosuppression. To minimize this risk, children will be dosed and prescribed hydroxyurea per standard institutional hydroxyurea guidelines and their clinician’s discretion. These guidelines recommend assessing self-reported adherence to hydroxyurea and obtaining blood work at least every three months to monitor for myelosuppression so that the prescribed hydroxyurea dose can be adjusted accordingly. The PI will review all severe myelosuppression events during the study (defined as an absolute neutrophil count <500/µL^3^, thrombocytopenia <50,000 µL^3^, and/or hemoglobin <7 g/dL with an absolute reticulocyte count <50×10^3^/μL^3^) for relatedness and expectedness to the intervention and these events will also be independently reviewed by the DSMB.  4. *Vulnerable Subjects:* We will enroll 60 subjects (~30 adolescents and ~30 caregivers) on the pilot RCT. The rationale for including adolescents as that these individuals are gaining autonomy and disease self-management skills and this intervention could help to support them. |
|  |
| - - If applicable, indicate which procedures may have risks to the subjects that are currently unforeseeable. (Include what actions will be taken to minimize the risks.) |
| Not applicable. |
|  |
| - - If applicable, indicate which procedures may have risks to an embryo or fetus should the subject be or become pregnant. (Include what actions will be taken to minimize the risks.) |
| There are no risks if a caregiver who is enrolled becomes pregnant. Adolescent/adult patient participants who become pregnant will be withdrawn as they will stop hydroxyurea. |
|  |
| - - If applicable, describe risks to others who are not subjects. (Include what actions will be taken to minimize the risks.) |
| Children whose caregivers enroll and are ≥9 years will be required to assent to their caregiver’s participation since they will be involved in the intervention. |

# Potential Benefits to Subjects

|  |
| --- |
| This study has potential to improve hydroxyurea adherence and could result in improved health and quality of life. Participants may also become more aware of the impact that SCD may have on their or their children’s health. |

# Data Management and Confidentiality

| - - Describe the data analysis plan, including any statistical procedures or power analysis. |
| --- |
| *Steps Prior to Conducting Analyses* Means, medians, ranges, standard deviations, and descriptive measures for nonnormality will be computed for continuous variables, as well as frequencies for categorical variables for each cohort and group. Data values identified as outliers will be examined to determine if they are data entry errors, in which case they will be modified when possible. Outliers will be included unless substantial evidence is available for their deletion. Statistical model assumptions will be examined and appropriate alternatives (e.g. allowing for heterogeneous variances and transformations to approximately normality) will be employed as needed.  Plan for Handling Drop-outs Efforts will be made to minimize drop-outs; nevertheless, they may occur. Whenever possible, the reason for withdrawal will be documented. If participants who drop-out provide consent, minimal data (i.e. laboratory studies) will be collected nearest the time of the research variables of interest.  *Formal Analyses*  Aim 1 Analyses: Retention rates for each arm and during each study period will be calculated to inform if refinements to our study procedures or to VDOT are needed prior to the efficacy RCT and to inform the number of study sites and participants that will be needed. A multi-method analysis approach will be used to assess engagement. Means, medians, ranges, standard deviations will be computed for quantitative items. Open-ended items survey items will undergo thematic content analysis by at least two reviewers until saturation is reached and no new themes emerge. We anticipate that retained and highly adherent participants be satisfied with VDOT and feel supported by the Scene Health staff, as well as have app system usage data that show that they consistently submit VDOT videos and respond to Scene Health staff messaging.  Analyses Aim 2: A multi-method analysis approach will be used to explore adolescents’ and caregivers’ needs and preferences for longer-term adherence monitoring and intervention. Again, means, medians, ranges, standard deviations will be computed for quantitative items. Open-ended items will undergo thematic content analysis by at least two reviewers until saturation is reached and no new themes emerge. We anticipate that ongoing electronic adherence monitoring and intermittent contact to encourage will be acceptable and promote sustained adherence behavior change.  Sample Size and Goals: We will conduct a pilot RCT and enroll 60 subjects (≥20 caregivers and ≥20 caregivers). We hypothesize that ≥80% of VDOT randomized subjects will be retained and will remain engaged with VDOT during the intervention period. Our other goals are to: 1) enroll ≥66% of those who are approached to the run-in period, 2) randomize ≥80% of the those who enroll, 3) have ≥80% of the randomized subjects on each arm (≥20/arm and with a mix of adolescent and caregiver subjects) continue to use their EM during the six-month ongoing monitoring period. Achieving these goals will support our ability to successfully recruit and retain subjects and sustain participant engagement with VDOT but will not significantly reduce our prospective pool of participants for the efficacy RCT at our sites. |
|  |
| - - Describe the steps that will be taken to secure the data (e.g., training, authorization of access, password protection, encryption, physical controls, certificates of confidentiality, and separation of identifiers and data) during storage, use, and transmission. |
| a) Clinical and laboratory data on the children of the caregiver who enrolls will be abstracted from the electronic medical records. This data will be collected as de-identified and stored within the secured REDCap database.  b) Survey data will be collected electronically and stored as de-identified data within the secured REDCap database  c) EM adherence data will be collected electronically from participants devices and stored as de-identified data on a secure electronic adherence device dashboard that is password protected and will only be accessible to research and Scene Health observation staff..  d) Subjects randomized to VDOT will submit daily videos of their children’s hydroxyurea administration using the secure Scene Health VDOT app. The Scene Health team will have access to the minimal identifiable subject information needed to administer the intervention (e.g., participant name, DOB, telephone number, and email). The Scene Health platform has an established HIPAA compliant mobile app. This system uses virtual communication (e.g. asynchronous video technology and secure 2-way in-app messaging) to support and encourage adherence through timely feedback and positive reinforcement. The system allows for secure, temporary storage of encrypted videos on smartphones until a network connection is made to a secure server. Access to the Scene Health app is protected by a secure username/password, face ID, or PIN login. Videos are not viewable in a phone’s general media galleries. If a device with the Scene Health app were lost or stolen, the Scene Health app itself could not be accessed without a username and password. Data sent from mobile devices are encrypted on the device as soon as it is collected. Patient's video data are deleted from the device as soon as receipt is confirmed by the server. Only approved project staff from the study sites and Scene Health will have access to patient data via the companion web portal.  e) Data on VDOT subjects use of the VDOT app may also be collected (e.g., number of times that they access the App, amount of time that they spend on the App daily, level of interaction with Scene Health staff through in-app chat) |
|  |
| - - Describe any procedures that will be used for quality control of collected data. |
| While the research study staff at each site will perform data entry for clinical and laboratory variables, the site PI will be responsible for verifying all clinical data that are entered into the secured REDCap database for each subject at their site. |
|  |
| - - Describe how data or specimens will be handled study-wide: |
| There are no study-specific specimens that will be collected as part of this study.  All participant videos that are submitted as part of the VDOT intervention and all electronic adherence data that is collected from participants’ electronic monitoring devices will be stored for at least 6 years. These videos and electronic monitor adherence data will be stored on the Scene Health and EM platforms, respectively. This server is HIPAA compliant. Videos will be deleted from the smartphone device as soon as receipt is confirmed by the Scene Health server to protect participant privacy. Only approved research staff from the study sites and Scene Health staff performing the VDOT observations will have access to patient data via the server’s secure and password protected web portal. |

# Provisions to Monitor the Data to Ensure the Safety of Subjects

| Data collection and accurate documentation will be the responsibility of the study staff under the supervision of the PI, Dr. Creary. Source documents and laboratory reports will be reviewed by the study team and data entry staff, who will ensure that they are accurate and complete. Study staff at the three sites will undergo a study training program on all procedures and how to abstract the data consistently at all three sites and the Nationwide Children's Hospital team will be available for any questions that may arise to ensure consistent data collection. The study team will meet at least biweekly to review the progress of the studies. The PI will review participant screening, accrual, attrition, adverse events, serious adverse events, unanticipated events, and protocol deviations at least monthly. The PI will ensure that all protocol deviations, adverse events, serious adverse events, and unanticipated problems are reported to the IRB and the NIH according to applicable regulatory requirements. Compliance of regulatory documentation and study data accuracy and completeness will be maintained through an internal study team quality assurance process.  Nationwide Children's Hospital will be the data coordinating site. Study data will be entered into REDCap, a secure, web-based application designed to support data capture for research studies. The REDCap databases will be created to minimize data entry errors with alerts embedded for items left blank or entries that are outside of an acceptable range for a given variable. The database incorporates an electronic audit trail to show change(s) to data after original entry including the date/time and user making the change. Reports will be generated by study staff on a monthly basis and discussed with the PI to monitor data quality. Data quality control checks will be performed on 10% of files by the PI or the PI’s designee. Study staff will be observed in their data collection activities annually by the PI or their designee to ensure study procedures are adhered to.  Patients who enroll or whose caregiver enrolls on this study will continue to be monitored as per standard of care during the study. While mild myelosuppression is expected and intended with hydroxyurea treatment, children on hydroxyurea occasionally need to temporarily suspend hydroxyurea and/or have their dose lowered to prevent severe myelosuppression and the complications that can result from severe myelosuppression (e.g., infection, bleeding, severe anemia). There is a potential risk that increased hydroxyurea exposure from increased hydroxyurea adherence could result in severe myelosuppression. To minimize this risk, patients will be prescribed hydroxyurea per their institutional hydroxyurea dosing guidelines and clinicians’ discretion. These guidelines recommend that clinical providers assess patients’ adherence to hydroxyurea and obtain blood work at least every three months so that their hydroxyurea dose can be adjusted accordingly. The PI will review all severe myelosuppression events during these studies (defined as an absolute neutrophil count <500/µL^3^, thrombocytopenia <50,000 µL^3^, and/or hemoglobin <7 g/dL with an absolute reticulocyte count <50×10^3^/μL^3^) for relatedness and expectedness and these events will also be independently reviewed by the DSMB. |
| --- |

# Provisions to Protect the Privacy Interests of Subjects

|  |
| --- |
| 1. Use of a secure database to store de-identified participant and patient data 2. The Scene Health app that will be used to deliver VDOT to participants has a data security system to ensure patient and data safety. The Scene Health platform has an established HIPAA compliant mobile app. This system uses virtual communication (e.g. asynchronous video technology and secure 2-way in-app messaging) to support and encourage adherence through timely feedback and positive reinforcement. The Scene Health system allows for secure, temporary storage of encrypted videos on smartphones until a network connection is made to a secure server. Access to the Scene Health app is protected by a secure username/password, PIN, or face ID login. Videos are not viewable in a phone’s general media galleries. If a device with the Scene Health app were lost or stolen, the Scene Health app itself could not be accessed without a username and password. Data sent from mobile devices are encrypted on the device as soon as it is collected. Patient's video data are deleted from the device as soon as receipt is confirmed by the server. Only approved project staff from the study sites and Scene Health will have access to patient data via the companion web portal. 3. The EM devices that will be used to store electronic adherence data have a secure platform. These data will only be stored via study ID number and accessible to the Scene Health and study team. |

# Data and Specimen Banking

|  |
| --- |
| Not applicable. |

# Sharing of Results with Subjects

|  |
| --- |
| Individual survey results and adherence data will not be shared directly with participants. Overall study results may be shared with participants via study newsletters or similar mechanisms. |

# Recruitment Methods

|  |
| --- |
| The PI at Nationwide Children’s Hopital and the Co-I’s at Lurie Children’s Hospital and Hasbro Children’s Hospital are pediatric hematologists. Multiple strategies will be used to identify and recruit prospective participants, including performing queries of clinical SCD databases and having an advertisement page available for clinic and research staff to pass out to patients and families with basic study information. Prospective participants will be approached during their routine hematology appointments, non-hematology appointments, and hospitalizations. If a prospective participant has an upcoming scheduled appointment, we may attempt to call them ahead of this visit to inform them that they may be eligible and that coming early for their clinical appointment could facilitate enrollment in this study. After confirming eligibility and reviewing study procedures, a research staff member will obtain informed consent and assent, if applicable. All enrolled participants will be assigned a study identification number. |

# Process to Document Consent in Writing

|  |
| --- |
| If a patient or caregiver meets eligibility criteria and agrees to participate, written documentation of consent will be obtained, as well as written documentation of assent (when applicable). We will be following the SOP: Written Documentation of Consent. |

# Consent Process

|  | |
| --- | --- |
| Consent (and assent, if applicable) will be obtained. Participants will be consented during their routine appointments (hematology and non-hematology) or when they are hospitalized. We will be following the SOP; Informed Consent Process for Research. | |
|  | |
| **Considerations for Consent Process** | |
| ***Waiver or Alteration Consent Process (consent will not be obtained, required information will not be disclosed, or the research involves deception)*** | |
| Not applicable. | |
|  | |
| ***Waiver of Written Documentation of Consent (verbal, virtual, and/or eConsent without signature)*** | |
| Not applicable. | |
|  | |
| **Non-English Speaking Subjects- (if not known, skip)**   - Indicate what language(s) other than English are understood by prospective subjects or representatives. - If subjects who do not speak English will be enrolled, describe the process to ensure that the oral and written information provided to those subjects will be in that language. Indicate the language that will be used by those obtaining consent. | Ability to speak English is an eligibility criteria for the study. |
| **Subjects who are not yet adults (infants, children, teenagers)**   - Describe the criteria that will be used to determine whether a prospective subject has not attained the legal age for consent to treatments or procedures involved in the research under the applicable law of the jurisdiction in which the research will be conducted. (E.g., individuals under the age of 18 years.)   - - - - For research, conducted in the state, review “SOP: Legally Authorized Representatives, Children, and Guardians (HRP-013)” to be aware of which individuals in the state meet the definition of “children.”         - For research, conducted outside of the state, provide information that describes which persons have not attained the legal age for consent to treatments or procedures involved the research, under the applicable law of the jurisdiction in which research will be conducted. One method of obtaining this information is to have a legal counsel or authority review your protocol along the definition of “children” in “SOP: Legally Authorized Representatives, Children, and Guardians (HRP-013).”       - Describe whether parental permission will be obtained from:         - Both parents unless one parent is deceased, unknown, incompetent, or not reasonably available, or when only one parent has legal responsibility for the care and custody of the child.         - One parent even if the other parent is alive, known, competent, reasonably available, and shares legal responsibility for the care and custody of the child.       - Describe whether permission will be obtained from individuals other than parents, and if so, who will be allowed to provide permission. Describe the process used to determine these individuals’ authority to consent to each child’s general medical care.       - Indicate whether assent will be obtained from all, some, or none of the children. If assent will be obtained from some children, indicate which children will be required to assent.       - When assent of children is obtained describe whether and how it will be documented. | For those <18 years of age, consent to participate will be required from one legal guardian. Legal guardianship is indicated within each child’s medical record and this will be reviewed prior to the consent/assent process.  The age of assent in all states that will be participating in this research is ≥9 years of age. Caregivers of children ages 1-10 years of age at enrollment will be the participants. However, the child of a caregiver who enrolls and is 9-10 years old will be required to provide written assent to their caregiver’s participation since they will be involved in the intervention. Also, we will obtain written assent for adolescent participants ages 11-17 years. |
| **Cognitively Impaired Adults**   - Describe the process to determine whether an individual is capable of consent. The IRB allows the person obtaining assent to document assent on the consent document and does not routinely require assent documents and does not routinely require cognitively impaired adults to sign assent documents. | When the clinical team reviews the clinical database to identify prospective participants, they will identify patients who are not cognitively capable of providing consent and the research team will not approach these subjects. |
| **Adults Unable to Consent**   - List the individuals from whom permission will be obtained in order of priority. (E.g., durable power of attorney for health care, court appointed guardian for health care decisions, spouse, and adult child.).   - - - - For research conducted in the state, review “SOP: Legally Authorized Representatives, Children, and Guardians (HRP-013)” to be aware of which individuals in the state meet the definition of “legally authorized representative.”         - For research conducted outside of the state, provide information that describes which individuals are authorized under applicable law to consent on behalf of a prospective subject to their participation in the procedure(s) involved in this research. One method of obtaining this information is to have a legal counsel or authority review your protocol along the definition of “legally authorized representative” in “SOP: Legally Authorized Representatives, Children, and Guardians (HRP-013).”       - Describe the process for assent of the subjects. Indicate whether:         - Assent will be required of all, some, or none of the subjects. If some, indicate which subjects will be required to assent and which will not.         - If assent will not be obtained from some or all subjects, an explanation of why not.         - Describe whether assent of the subjects will be documented and the process to document assent. The IRB allows the person obtaining assent to document assent on the consent document and does not routinely require assent documents and does not routinely require subjects to sign assent documents. | Adult patients and legal guardian caregivers who are unable to provide consent will not be eligible to participate.  The child of a caregiver who enrolls and is ≥9 years will be required to provide written assent to their caregiver’s participation since they will be involved in the intervention. Also, we will obtain written assent for adolescent participants ages 11-17 years. |
| **Consent for HUD**   - For HUD uses provide a description of how the patient will be informed of the potential risks and benefits of the HUD and any procedures associated with its use. | Not applicable |

# Compensation for Research-Related Injury

|  |
| --- |
| This study does not involve more than Minimal Risk to subjects. |

# Compensation to Subjects

|  |
| --- |
| All subjects who are randomized will receive a smartphone and unlimited data plan to use during the study.  All participants will receive $20 for completing each study visit.  Participants who are randomized to VDOT will also be eligible to receive compensation as part of the VDOT intervention if they meet the 90% or higher adherence threshold ($30) or increase their adherence by at least 10% ($15) |

# Economic Burden to Subjects

|  |
| --- |
| We do not anticipate any additional costs to participants or their families because of participation in the research study. Study visits will coincide with clinical visits wherever possible and after the randomization visit, study visits will be able to be completed electronically, if needed, to limit burden to subjects. |

# Setting

|  |
| --- |
| The PI at Nationwide Children’s Hospital and the Co-I’s at Lurie Children’s Hospital and Hasbro Children’s Hospital are pediatric hematologists. Multiple strategies will be used to identify and recruit prospective participants, including performing queries of clinical SCD databases and having an advertisement page available for clinic staff to pass out to patients and families with basic study information. Prospective participants will be approached during their routine hematology appointments, non-hematology appointments, and hospitalizations. If a prospective participant has an upcoming scheduled appointment, we may attempt to call them ahead of this visit to inform them that they may be eligible and that coming early for their clinical appointment could facilitate enrollment in this study. Research study procedures will be completed either in the clinical setting or participants will also be able to complete many of the assessments electronically at home. |

# Resources Available

|  |
| --- |
| The PI and Co-I’s on this study are all pediatric hematologists who provide care to the population of children and young adults who will be (or whose caregiver will be) recruited. Each site will have at least one research associate dedicated to recruitment and data collection and these study staff will be expected to review this protocol. The PI, Co-Is and research coordinators will meet regularly to review the progress of the study and to discuss any challenges with the research, the research procedures. Members from the Scene Health observation team may also be asked to attend these meetings intermittently to ensure clarity about the protocol and procedures, duties and functions.  Nationwide Children's Hospital, Hasbro Children’s Hospital, and Lurie Children’s Hospital are all pediatric tertiary hospitals that follow 100-200 patients (approximately 50% children and 50% adolescents) with SCD who meet (or whose caregiver meets) eligibility criteria.  We anticipate having to approach ≤90 prospective subjects (≤30/site) to recruit our planned total sample of 60 subjects (with ≥20 of these subjects being caregivers and ≥20 of these subjects being adolescents) to the run-in period. We anticipate that we will be able to recruit these subjects in six months, which assumes a similar accrual rate at each site to the previous VDOT study. |

# Confidentiality of Data Collection

| - - How long will identifying information on each participant be maintained? (Study data and identifiers must be stored for at least six years after study closure; a longer period may be used. A specific date or number of years must be listed.) |
| --- |
| Study data and identifiers will be stored for at least six years after study closure. |
|  |
| - - Describe any plans to code identifiable information collected about each participant. |
| All enrolled participants will be assigned a study identification number. All clinical, laboratory, and survey data will be kept secure and de-identified and coded by study identification number, rather than by personal identifiers to prevent confidentiality breaches.  An identifiable subject list coded by study ID will be kept separate from de-identified data to track enrollment and key study milestones. This list will be the only link to de-identified data and will be securely stored on the hospital’s protected research drive which is only accessible by approved study staff. |
|  |
| - - Check each box that describes steps that will be taken to safeguard the confidentiality of information collected for this research:   x Research records will be stored in a locked cabinet in a secure location  x Research records will be stored in a password-protected computer file  x The list linking the assigned code number to the individual subject will be maintained separately from the other research data  x Only certified research personnel will be given access to identifiable subject information.  x Scene Health study staff will have access to the videos that have full-views of the participants randomized to VDOT but will only be given the minimal identifiable subject information needed to facilitate the intervention (e.g., telephone number, email address, participant name, and participant DOB). . |
| - - Describe the **provisions included in the protocol to protect the privacy interests of subjects, where "privacy interests" refer to the interest of individuals in being left alone, limiting access to them, and limiting access to their information. (This is not the same provision to maintain the confidentiality of data.)** |
| Participants privacy will be ensured by only contacting participants as frequently as outlined in the study procedures and by the intervention.  While the research team may need to reach out to participants to complete study measures, they will limit the number of these attempts that they make so that participants right to be left alone is maintained. |
|  |
| - - **Will it be necessary to record information of a sensitive nature?**   “Sensitive information includes but is not limited to ‘information relating to sexual attitudes, preferences, or practices; information relating to the use of alcohol, drugs, or other addictive products; information pertaining to illegal conduct; information that, if released, might be damaging to an individual's financial standing, employability, or reputation within the community or might lead to social stigmatization or discrimination; information pertaining to an individual's psychological well-being or mental health; and genetic information or tissue samples.’”  Yes  No |
| - - **Do you plan to obtain a federally-issued Certificate of Confidentiality as a means of protecting the confidentiality of the information collected? (If the study is NIH funded, Certificate of Confidentiality will be issued; please mark “Yes”).**   “Certificate of Confidentiality (Certificate) protects the privacy of research participants enrolled in biomedical, behavioral, clinical, or other types of health-related research that collect or use identifiable, sensitive information. With limited exceptions, researchers may not disclose names or any information, documents or biospecimens containing identifiable, sensitive information. The Certificate prohibits disclosure in response to legal demands, such as a subpoena.”  Yes  No |

# Protected Health Information (PHI) Recording

| *PHI: individually identifiable health information transmitted or maintained in any form (electronic means, on paper, or through oral communication) that relates to the past, present or future physical or mental health or conditions of an individual plus any of the following 18 identifiers.* |
| --- |
| Are you accessing or recording information related to the provision of healthcare or payment for healthcare services (medical records, lab test results, diagnoses, etc.)?  Consider sources such as the medical record, clinic schedules, billing statements, etc. This includes screening for potential subjects for recruitment purposes.  If yes, complete section 28.0.  If no, skip section 28.0. |
| Indicate which subject identifiers will be ACCESSED for this research.   \| Name  Address *(including street, city, state, zip code and county)*  Dates *(treatment dates, birth date, date of death)*  Telephone  Fax Number  Email address,  Social Security Number *(do not check if only used for ClinCard)*  Medical Record Number or other account number (specify below)  Health Plan Beneficiary Identification Number \| Account Numbers  Certificate/License Numbers  Vehicle Identifiers and Serial Numbers  Device Identifiers and Serial Numbers  URL  IP address  Biometric identifiers, including finger and voice prints  Full face photographic images and/or any comparable images  Other number, characteristic or code that could be used to identify an individual (specify below)  None *(Complete De-identification Certification Form)* \| \| --- \| --- \| \|  \| \| \| If the “Medical Record Number or other account number) and/or “Other number, characteristic or code that could be used to identify an individual” please respond below. \| \| \| Medical record number will be used only. \| \|   Indicate which subject identifiers will be RECORDED for this research.   \| Name  Address *(including street, city, state, zip code and county)*  Dates *(treatment dates, birth date, date of death)*  Telephone  Fax Number  Email address,  Social Security Number *(do not check if only used for ClinCard)*  Medical Record Number or other account number (specify below)  Health Plan Beneficiary Identification Number \| Account Numbers  Certificate/License Numbers  Vehicle Identifiers and Serial Numbers  Device Identifiers and Serial Numbers  URL  IP address  Biometric identifiers, including finger and voice prints  Full face photographic images and/or any comparable images  Other number, characteristic or code that could be used to identify an individual (specify below)  None *(Complete De-identification Certification Form)* \| \| --- \| --- \| \|  \|  \| \| If the “Medical Record Number or other account number) and/or “Other number, characteristic or code that could be used to identify an individual” please respond below. \| \| \| Medical record number will be used (not other account number).  Full face videos of participants and the children of caregiver participants taking hydroxyurea who are randomized to VDOT will also be sent to our observation team at Scene Health. See provisions above documenting how these data will be protected. \| \| |

# Confidential Health Information Recording

| Please mark all categories that reflect the nature of health information to be ACCESSED and USED as part of this research.   \| Demographics *(age, gender, educational level)*  Diagnosis  Laboratory reports  Radiology reports  Discharge summaries  Procedures/Treatments received  Billing information \| Names of drugs and/or devices used as part of treatment  Location of treatment  Name of treatment provider  Surgical reports  Other information related to course of treatment  None \| \| --- \| --- \| |
| --- | --- | --- |
| If the information you are collecting does not match any of the options listed above, please list what information you will be gathering. |
| Not applicable. |

# Waiver or Alteration of HIPAA Research Authorization

| - - **Check the appropriate category and attach the required form on the Local Site Documents, #3. Other Documents, page of the application.  (Mark all that apply.)**   Protocol meets the criteria for waiver of authorization. (Continue filling out section 30.)  Patient Authorization will be obtained. (Include the appropriate HIPAA language (see Section 14 of consent template) in the consent form OR attach the [HRP-900, HIPAA AUTHORIZATION](http://eirb2.nationwidechildrens.org/IRBProd/sd/Rooms/DisplayPages/LayoutInitial?container=com.webridge.entity.Entity%5BOID%5BE85E349BFA61434CBF51F4CB8B91D7E6%5D%5D&tab2=294B1E5FDD6A8142A2AF57F5F7F197BF) form.)  Protocol is using de-identified information. (Attach the [HRP-902, DE-IDENTIFICATION CERTIFICATION](http://eirb2.nationwidechildrens.org/IRBProd/sd/Rooms/DisplayPages/LayoutInitial?container=com.webridge.entity.Entity%5BOID%5BE85E349BFA61434CBF51F4CB8B91D7E6%5D%5D&tab2=294B1E5FDD6A8142A2AF57F5F7F197BF) form.)  Protocol involves research on decedents. (Attach the [HRP-903, RESEARCH ON DECEDENTS REQUEST](http://eirb2.nationwidechildrens.org/IRBProd/sd/Rooms/DisplayPages/LayoutInitial?container=com.webridge.entity.Entity%5BOID%5BE85E349BFA61434CBF51F4CB8B91D7E6%5D%5D&tab2=294B1E5FDD6A8142A2AF57F5F7F197BF) form.)  Protocol is using a limited data set and data use agreement. (Contact the Office of Technology Commercialization to initiate a Limited Data Use Agreement.)  **Find the HIPAA forms in the eIRB2 Website Library, Templates.** |
| --- |
|  |
| - - Please indicate the type of waiver or alteration needed for this study.   *Note: If the research involves more than one type of waiver, check the appropriate box.*  A. Partial Waiver (to access PHI for recruitment purposes only)  B. Full Waiver (entire research study)  C. Alteration (waiver of written documentation of authorization; obtaining verbal authorization to use PHI) |
|  |
| - - Please discuss why it is necessary to access and review the health information noted in your response above. |
| Medical information will be accessed and reviewed to determine eligibility. To evaluate the impact of VDOT on laboratory and clinical outcomes, we will need to access and review health information of the adolescent participants and the children of the caregiver participants. Accessing and reviewing health information will also limit burden on participants to self-report some of the demographic data we will collect. |
|  |
| - - Is the health information to be accessed and reviewed the minimal necessary to achieve the goals of this research? Please provide an explanation, as well. |
| Yes  No |
| Yes. We will only access and review health information that is necessary to determine the impact of our intervention on laboratory and clinical outcomes and also to limit participant burden. |
|  |
| - - Explain how the access, use, or disclosure of PHI presents no more than a minimal risk to the privacy of the individual. |
| The potential risk of accessing, using, or disclosing PHI is loss of confidentiality which is no more than a minimal risk to the privacy. |
|  |
| - - Describe your plan to protect the identifiers (or links to identifiable data) associated with the PHI from improper use and disclosure, including where PHI will be stored, what security measures will be applied, and who will have access to the information. Describe the safeguards for electronic and/or hard copy records. |
| -Research records will be stored in a locked cabinet in a secure location  -Research records will be stored in a password-protected computer file  -To maintain confidentiality and also blinding of the investigators, the list linking the assigned code number to the individual subject will be maintained separately from the other research data  -Only certified research personnel will be given access to identifiable subject information.  -Members of the Scene Health team who will deliver the intervention to those randomized to VDOT will have access to the videos that participants submit but only be given access to the minimal identifiable subject information needed to facilitate the intervention (e.g., telephone number, email address, participant name, and participant DOB)  -EM devices will store adherence data by study ID number only and will not have access to identifiable participant information. |
|  |
| - - Will identifiers (or links to identifiable data) be destroyed? Provide a justification for your answer. |
| Yes – Describe the plan to destroy the identifiers at the earliest opportunity consistent with the conduct of the research. Include **when** (consistent with section 27) and **how** identifiers will be destroyed.  No – Provide the legal, health, or research justification for retaining the identifiers. Legal justification should include a brief description/citation of the legal requirement.  N/A – Will not record identifiers or create links or codes to connect the data. |
| Study identifiers will be stored for at least six years after study closure and will be deleted from the password protected and separate database that will store them. |
|  |
| - - Explain why a waiver/alteration (instead of written authorization) is needed to conduct the research. |
| NA |
|  |
| **NOTE: Only those personnel listed on the IRB-approved application may access PHI and medical information.**  *Reminder: Protected Health Information obtained as part of this research will not be reused or disclosed to any other person or entity other than those listed (except as required by law for authorized oversight of the research project) without additional approval. IRB/Privacy Board approval must be obtained for other research involving the use or disclosure of this PHI.* |

# Multi-Site Research

| *****This section is required when research will be conducted at multiple sites. Check the appropriate box.***  NCH **IS** participating in multi-site research.  NCH **IS NOT** participating in multi-site research. No further information is needed in this section. |
| --- |
| - - TOTAL Number of Subjects to be Enrolled Study-Wide   - List the locations of the additional sites. Please indicate which sites are International. |
| Total number of subjects to be enrolled study-wide:  60  Locations:  Nationwide Children’s Hospital Columbus, OH  Hasboro Children’s Hospital Providence, RI  Lurie Children’s Hospital Chicago, IL |
|  |
| - - Study-Wide Recruitment Methods |
| See section 19.0. Recruitment methods will be the same across sites. |
|  |
| - - Multi-Site Data Management   - Check all applicable boxes below.   NCH will SEND data to other sites  NCH will RECEIVE data from other sites   - - Describe the procedures for maintenance or confidentiality of data at non-NCH sites. |
| No specimens will be received from other sites but NCH will receive data from other sites.  Study data will be stored for at least 6 years after study closure. Only certified research personnel will be given access to the data. The PI (Creary) and Co-Is (McGann and Badawy) will be responsible for receipt and transmission of the data. Data will be transported electronically using a secure database (RedCap). |
|  |
| NCH investigator **IS** the LEAD investigator in the study.  NCH **IS NOT** the LEAD investigator in the study. No further information needed in this section. |
| Nationwide Children's Hospital will be responsible for conducting the scientific review for this study and ensuring the concordance between grants and the IRB application.  Each of the three study sites will be responsible for confirming with their own IRB that their study staff have completed relevant trainings and are qualified to conduct the research and reviewing non-compliance/study protocol deviations.  The two relying sites (Hasboro and Lurie Childrens’ Hospitals) will provide ensure that consent forms meet institutional language and HRPP requirements and will prepare and submit study materials for continuing review to Nationwide Children's Hospital for sIRB review. |
